# An EHR-based method to structure, standardize, and automate clinical documentation tasks for pharmacists to generate extractable outcomes

**DOI:** 10.1093/jamiaopen/ooad034

**Published:** 2023-05-11

**Authors:** Kimberly A Sanders, Daniel Wolverton, Marina Stamopoulos, Rada Zunich, Joshua Niznik, Stefanie P Ferreri

**Affiliations:** Division of Practice Advancement and Clinical Education, UNC Eshelman School of Pharmacy, Chapel Hill, North Carolina, USA; Department of Pharmacy, UNC Health, Chapel Hill, North Carolina, USA; Department of Pharmacy, UNC Health, Chapel Hill, North Carolina, USA; Division of Practice Advancement and Clinical Education, UNC Eshelman School of Pharmacy, Chapel Hill, North Carolina, USA; Division of Geriatrics and Center for Aging and Health, Division of Pharmaceutical Outcomes and Policy, UNC School of Medicine, Chapel Hill, North Carolina, USA; Division of Practice Advancement and Clinical Education, UNC Eshelman School of Pharmacy, Chapel Hill, North Carolina, USA

**Keywords:** deprescriptions, electronic health records, primary health care, research design, aged, clinical pharmacy information systems

## Abstract

As the recognition of team-based care grows and pharmacists increase in patient care interventions, it is important that tools to track clinical services are easily accessible and well-integrated into workflow for all providers. We describe and discuss feasibility and implementation of data tools in an electronic health record to evaluate a pragmatic clinical pharmacy intervention focused on deprescribing in aged adults delivered at multiple clinical sites in a large academic health system. Of the data tools utilized, we were able to demonstrate clear documentation frequency of certain phrases during the intervention period for 574 patients receiving opioids and 537 patients receiving benzodiazepines. Although clinical decision support and documentation tools exist, they are underutilized or cumbersome to integrate into primary health care and strategies, such as employed, are a solution. This communication incorporates the importance of clinical pharmacy information systems in research design.

## BACKGROUND AND SIGNIFICANCE

Healthcare professionals recognize the value of an interprofessional approach to patient care, and now there are ways to document these interprofessional recommendations within an electronic health record (EHR). However, many of these clinical decision documentation tools are often underutilized due to additional steps to the workflows. Healthcare professionals also do not like using systems developed outside the main EHR because of lack of integration (known as “double documenting”).^1^ There is a specific need for pharmacists involved in healthcare teams to document and track clinical services and interventions in a way that is easily accessible and well-integrated into clinical workflows for other providers.^2^

Involving pharmacists on the team optimizes patient care and decreases practitioner task-load by shifting certain responsibilities to the pharmacist, such as chronic disease management and therapeutic drug monitoring.^3–5^ Despite evidence showing the benefits of involving pharmacists, healthcare systems have not adapted to including them on teams in a consistent manner. As pharmacists take on more responsibility with patient care, there are several challenges pharmacists face with documentation in the EHR, including time constraints, significance or appropriateness of documentation, and utility for tracking productivity and outcomes of pharmacist work that need to be overcome.^1^ All of these challenges can also fuel pharmacist burnout and lack of engagement. By providing standardized, trackable processes to overcome these challenges, healthcare systems can improve evaluating ways to capture the value of pharmacists’ intervention, increase their involvement within interprofessional teams, and generate useful clinical and process-related data.

Documentation tools generally available for tracking medication changes and pharmacist interventions that are integrated into the EHRs are presented in Table 1.^6–15^ Current tools available include *SmartPhrases* (preset phrases), *SmartLists (*dropdowns), *SmartForms* (template), and *iVents* (intervention forms). Since the tools used are proprietary to a specific EHR, naming and availability of the tools may vary between different systems. Throughout the rest of the paper these data tools will be referred to by the following terminology. Preset phrases are shortcuts for entering text and allow text to be inserted into the EHR by typing an abbreviated phrase. The phrases can be words to entire note templates or other EHR information like labs. Dropdowns allow the user to select from a list of common choices that are predetermined. These dropdowns can be contained within the preset phrases. Templates capture discrete data and are highly customizable with specific areas to address during the encounter. Users click through predetermined questions that can be tracked. Finally, intervention forms are generally utilized by pharmacists to document medication-related problems such as adverse effects, adherence/cost concerns, and others. Some examples of screenshots of how the dropdown feature and the template for consult notes appear and were used for study purposes have been included in Supplementary Materials. Data collected by these tools can be tracked and extracted alongside patient-level EHR data in order to capture pharmacist and other healthcare provider interventions and evaluate their impact on outcomes.

**Table 1.** Tools for tracking medication changes and pharmacist intervention within an EHR

Tool	Description	Strengths	Limitations
*Template (eg, SmartForms)*	Clinical workflow tool for organized review of specific conditions; allows for effective and efficient data capture, documentation within a clinical visit, and integrated, actionable decision support in a single environment^6^	(1) Supports clinical decisions by containing a series of actions/responses to questions in the form that can turn responses into a note format (2) Data selected within form is coded and reportable	(1) Potentially labor intensive as each disease state requires its own form development and lacks integration tying multiple disease states together (2) Disease-specific prevention-related reminders only present on patient summary page
*Dropdown (eg, SmartLists)*	Checklist organized in clinically relevant sections; each filtered item provides a discrete list of options that the user selects from^7^^,^^8^	(1) Has ability to drive decision making and create action/order in EHR by serving as a checklist (2) List can extract data from EHR in real time	(1) Potentially intrusive to provider autonomy due to limiting predetermined choices (2) May be difficult to use, leading to decreased efficiency
*Preset Phrases (eg, SmartPhrases)*	Consists of a section of text intended to be inserted quickly into an EHR progress note using an abbreviated phrase; may contain both prepopulated default text and areas requiring completion before the note can be signed.^9^	(1) Prepopulated sections to optimize decision making and efficiency (2) Generally easy to use, implement, and track usage over time	(1) Indirect costs for developing and monitoring EHR-based reports through data analysts (2) More complex and reportable phrases require assistance of EHR support staff
*Intervention Forms (eg, iVents)*	Documents pharmacist intervention (ie, med change, dose adjustment, symptom management, education, etc.), to create a more standardized follow-up process between pharmacists and providers on interdisciplinary teams^11–14^	(1) Ability to track interventions and return to opened interventions (2) Standardized phrasing reduces hand-off time and discrepancies/confusion	(1) Varying documentation practices or underdocumentation and lack of consistency between health record systems (2) Clinical focus—no financial or administrative component

## OBJECTIVE

The objective of this communication is to provide an example of an innovative application of documentation data tools in an EHR at a large academic medical center as a standardized method to track pharmacist interventions as part of a deprescribing trial in outpatient primary care clinics.

The application of preset phrases and dropdowns described below was part of an ongoing pragmatic randomized study of a pharmacist intervention to deprescribe opioids and benzodiazepines (BZDs) for older adults seen in primary care practices in North Carolina (registered at clinicaltrials.gov [NCT04272671]).^15^ Briefly, the objective of the study was to evaluate the effectiveness of a clinical pharmacist intervention to identify patients aged ≥65 years old at risk for falls, based on chronic opioid and/or BZD use, and subsequently provide targeted deprescribing recommendations to primary care prescribers to help initiate tapering plans. A medication tapering plan or “taper” helps a patient to safely discontinue a medication to reduce the risk of withdrawal symptoms from dependency and adverse outcomes after a medication is deprescribed by a provider. It includes incremental reductions in the dosage over time until it is zero. Opioids and BZDs are 2 major classes of medications that are historically difficult to deprescribe in older adult populations. As part of the study, the study team sought to collect data on intervention delivery, including pharmacist screening for eligibility, types of recommendations, and whether recommendations were accepted.

## MATERIALS AND METHODS

### Development of workflow functionality

**Figure 1.**
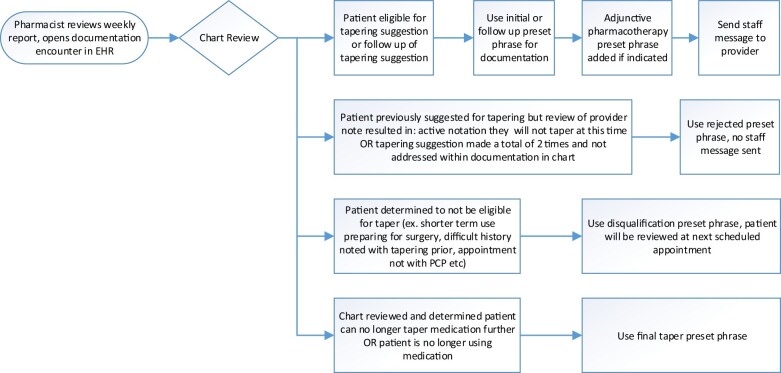
Pharmacists workflow.

The target population was older adults who were receiving chronic opioids and/or BZDs, defined as at least 4 prescriptions for either an opioid or BZD in the last year and at least 1 prescription in the last 90 or 180 days, respectively. An automated weekly report (generated outside the EHR) was used to identify patients who had an upcoming clinic visit. The report filtered out patients based on exclusion criteria of active chemotherapy (or radiation) within the last year, a diagnosis of dementia, non-English speaking, or upcoming visit with a provider who was not capable of making changes to prescriptions for controlled substances (eg, lab, nursing). The general workflow for pharmacists conducting the chart reviews is presented in Figure 1. Briefly, the pharmacist would review the report and conduct a brief chart review to determine the appropriate note template to use. Based on this review, the pharmacist would open a documentation encounter and proceed to use the different data tools described. The notes entered were not visible to the patient but were part of the patients overall permanent medical record.

### Development of data tools functionality

**Figure 2.**
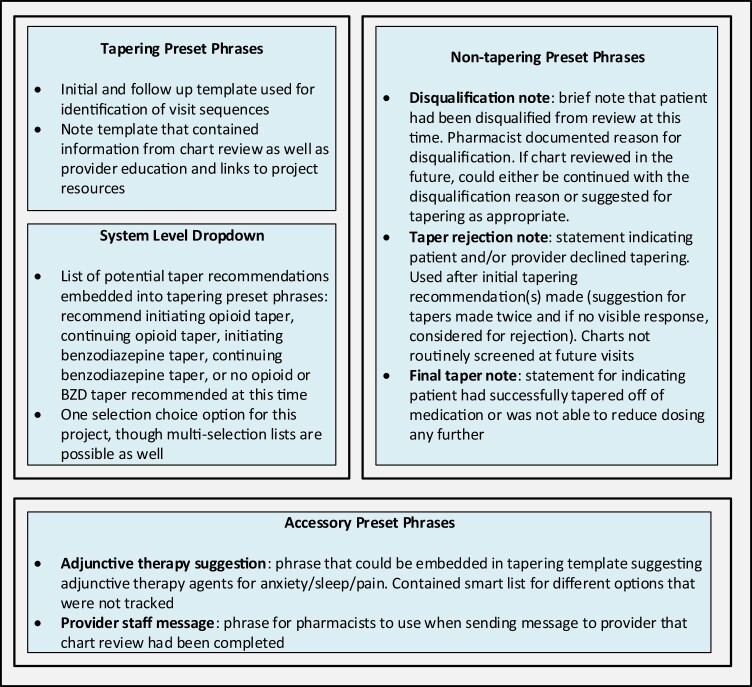
Summary of data tools used in project.

For tracking pharmacist interventions, a combination of tools within the EHR were used. One tool was a system dropdown list. This list contained 5 options and allowed for the study team to determine the pharmacist recommendation for the patient based on the selected statement. The clinical pharmacy team partnered with EHR analysts within the health system to build this list, meaning to configure the EHR to have the list readily available within the system. The dropdown list was named specifically for the department and was only to be used within the preset phrases explained below in Figure 2. This helped verify that reported data was solely from the research project on deprescribing.

Other tools included user-developed note templates using preset phrases. Initially the project was designed with 3 named preset phrases: initial recommendations, follow-up recommendations, and disqualification from chart review. A few months into the chart review process, 2 additional preset phrases were added (final taper and rejected taper). Accessory phrases were also developed. These note templates were developed by pharmacists doing the chart reviews and shared with other team members without having to go through the EHR team (see Supplementary Materials). For the purposes of the research project, the initial and follow-up note templates were identical with the only change being the name of the preset phrase used in the chart to allow for evaluation of extractable implementation and process outcomes. By tracking the preset phrases, the data team was able to determine if the patient had been reviewed by the pharmacist previously (initial versus follow-up notes), what the specific recommendations of the pharmacist were (using the dropdown lists and preset phrase if an adjunctive therapy was suggested), and if the patient had reached an endpoint (using final or rejected preset phrases). Application to our study design and analysis is described below.

## RESULTS

### Evidence of utilization

As a result of the built features in the data tools deprescribing documentation, the study team was able to track in real-time how consultant pharmacists utilized the preset phrases and dropdown list recommendations. The data presented below is based on documentation during the intervention period for 574 patients who were receiving opioids and 537 patients who were receiving BZD. Preset phrases were not mutually exclusive and also included adjunctive therapy recommendations related to the conditions being treated (Table 2).

**Table 2.** Preset phrases used and patient recommendations

	Opioid *n* (%) of 574 patients	BZD *n* (%) of 537 patients
Preset phrases used (note templates)		
Consult note initial	469 (81.7%)	415 (77.3%)
Adjunctive therapy	332 (57.8%)	282 (52.5%)
Consult note follow-up	148 (25.8%)	127 (23.7%)
Final taper statement note	11 (1.9%)	14 (2.6%)
Disqualification	127 (22.1%)	117 (21.8%)
Rejected taper	238 (41.5%)	175 (32.6%)
Missing/none	49 (8.5%)	62 (11.6%)
Dropdown list items used (recommendations)		
Initiating opioid taper	401 (69.8%)	38 (7.1%)
Continuing opioid taper	58 (10.1%)	4 (0.7%)
Initiating BZD taper	57 (9.9%)	375 (69.8%)
Continuing BZD taper	17 (2.9%)	67 (12.5%)
No opioid or BZD taper recommended at this time	43 (7.5%)	21 (3.9%)
Missing/none	105 (18.3%)	122 (22.7%)

In addition to tracking intervention delivery and recommendations, preset phrases also helped inform the number of patients not meeting inclusion criteria and being disqualified from participation. As stated in the workflow, exclusion criteria was built into the automated screening reports to exclude certain visit types, which increased the efficiency of manual patient screening by the consultant pharmacists to determine study eligibility using the “disqualify” preset phrase. Documentation indicating rejected recommendations for tapering was used where prescribers reviewed a previous consult intervention note and determined not to act on the recommendations or indicated that they had different justification for why the patient should not proceed with a taper plan. The type of dropdown list recommendation was a way to designate next steps in the initial and follow-up consult intervention documentation (Table 2). Categorizing these allowed tracking of when a taper was indicated and for what type of medication. Missing recommendations occurred in situations when the patient was disqualified or the taper was rejected, so no recommendation was provided.

Initial consult notes were understandably the most frequently used preset phrase to initiate an intervention consult (81.7% and 77.3% of patients) and thus, initiating an opioid taper and/or benzodiazepine taper were the most frequently used dropdown list recommendations (69.8% and 69.8% of patients). The adjunctive therapy preset phrase was also used for more than half of patients, demonstrating the potential utility of recommendations other than initiating or continuing tapers. For example, a patient could have a recommended adjunctive therapy regardless of taper status in their consult note signifying the importance of evaluating nonnarcotic alternatives in treatment. Preset phrases for continuing tapers were used infrequently, due to either lack of follow-up visits during the study time frame or the process noted above where a patient was given 2 “initiation” taper notes before the suggestion was considered a “rejected” status (ie, no need for follow-up).

## DISCUSSION

### Plan/utilization for research and future developments

This project demonstrated that data tools can be incorporated into the workflow of a consultant clinical pharmacy service with a high degree of uptake. The project also demonstrated that these tools have great potential utility for measuring intervention delivery and extractable outcomes for pragmatic trials embedded within healthcare systems. We originally developed this process for data collection as a way to measure the fidelity of our intervention. Our intent was to measure the level of “adherence” to the intervention, based on whether the pharmacy team deemed patients ineligible for the study (ie, disqualified) or if the provider rejected the pharmacist tapering recommendation (ie, rejected). The standardized data tools used in this study were easily extractable and linkable to patient-level or encounter-level data for further study. As a result, we have been able to characterize and account for clinic-level variation, based on the proportion of recommendations that were accepted versus rejected and the proportion of patients that were deemed ineligible for the study, when evaluating the impact of our intervention. This has also enabled the study team the ability to conduct per-protocol or sensitivity analyses that exclude patients who were disqualified or those who had recommendations rejected to get a more accurate estimate of the effect of the intervention among those patients who actually received it. Finally, the easily extractable data outcomes from our workflow have proven useful as a potential method to track intentional deprescribing as an outcome in the EHR, rather than relying on gaps in medication orders or medication supply, which cannot differentiate between deprescribing and unintentional nonadherence, or otherwise conducting detailed and time-consuming chart reviews.

The benefits of this type of data allow for measurable impact of clinical interventions in a more accessible format with improved integration into the EHR for various healthcare professionals. Most of these EHR data tools are easy to create specifically for an intervention and share with the staff members completing the work. It took an estimated 2–3 h of development to create the preset phrases including getting feedback from the data team and revising the phrases. Creation of the dropdown list took several weeks to fully implement only because this required assistance from the EHR analysts. Getting the usage of the preset phrases within the EHR is simple but will require special access to a data reporting warehouse, and a standard end user of the EHR will likely not be able to access this information without assistance. Additionally, automating criteria allows for easier and real-time tracking as needed. Some challenges to keep in mind for utilization are that IT infrastructure are crucial for the feasibility. Also, there is potential for a growing number of preset phrases to manage depending on how detailed reporting needs are and so monitoring of the ones used to track impact are necessary.

### Limitations

One limitation noted during the study was that the dropdown created for the study intervention was a “system list,” meaning it is available to all users within the health system. Thus, it is possible that the same tools were used by providers external to the study to document for patients not included in the study. However, due diligence was done to name the list specifically for the department and only used in conjunction with preset phrases for the note templates. Also, some of the nontapering notes (such as the disqualification note) were not able to report the reason for disqualification consistently as asking for the reason for disqualification was not built into the note since it was posted to the patient’s permanent health record. This emphasizes the importance of the build/configuration within the EHR and intent of the phrases so that they collect all the important aspects of data to be tracked. More detailed tracking information through templates or other lists may have required larger IT involvement as well as increased oversight from system documentation committees.

## CONCLUSION

The application of data tools in the EHR to track medication changes and pharmacist interventions through extractable outcomes can be successfully used in practice for productivity monitoring and research for real-world clinical interventions.

## Supplementary Material

ooad034_Supplementary_Data

## Data Availability

The data underlying this article will be shared on reasonable request to the corresponding author.
