# Supplementary material for: An EHR-based method to structure, standardize, and automate clinical documentation tasks for pharmacists to generate extractable outcomes
Source: JAMIA Open. 2023 May 11;6(2):ooad034. doi: 10.1093/jamiaopen/ooad034 (PMC10174697; doi:10.1093/jamiaopen/ooad034)

**Supplemental Materials**

**Pharmacy Consult Note – Opioid/Benzodiazepine Deprescribing Recommendation (CDC Study, IRB#18-2920)**

@NAME@ is a @AGE@ @SEX@ who was identified as being on a long-term medication that puts them at an increased risk for falls

| **Recommendations**   - **1** - Consider initiating **2** *** as an adjunctive therapy for relief from *** - ******* (Free Text recommendations) |
| --- |

*** (tapering table inserted)

- Consider the addition of non-pharmacological therapies: **3; 4; 5**
- Recommend follow-up in *** {days/weeks/months} to assess symptoms, side effects, and patient adherence to tapering regimen.
- Tapering schedule may be slowed or paused if patient is experiencing inadequate symptom control or symptoms of withdrawal
- ***Recommend completion of the Ambulatory Falls Risk Assessment during next patient visit
- Consider implementing {Opioid or Benzodiazepine} taper agreement with the patient. Click the appropriate hyperlink that follows for printable agreement ([Opioid Taper Agreement](http://deprescribe.web.unc.edu/files/2020/04/Opioid-Taper-Agreement_UNC.pdf); [Benzodiazepine Taper Agreement](http://deprescribe.web.unc.edu/files/2020/04/Benzodiazepine-Taper-Agreement_UNC.pdf))

A-TAPER: UNC Guidance for Deprescribing Opioids and Benzodiazepines.

This website contains helpful resources for providers and patients:

<http://deprescribe.web.unc.edu/>

Please contact consult pharmacist with questions or clarifications:

***

This chart review was completed by @ME@, PharmD on @EDTODAYDATE@ @TIMESTAMP@ . The consultant pharmacist was not involved in the direct care of the patient. The information contained herein is a recommendation based in part on patient chart information, the current standard of care, and general practice guidelines. All final tapering decisions should be based on the provider's own clinical judgement.

| **Summary of Chart Review** |
| --- |

Patient taking the following **opioid(s):** {opioids list} *** for a total duration of **6**. Indication for use based on chart review: ***

Current MME/day (per PDMP) = ***

30 day average MME/day (per PDMP) = ***

Patient taking the following **benzodiazepine(s):** {benzodiazepine list} *** for a total duration of **6**. Indication for use based on chart review: ***

Current LME/day (per PDMP) = ***

30 day average LME/day (per PDMP) = ***

*****The consultant pharmacist has reviewed the NC Controlled Substances Reporting System and the patient appears to be using *** {consistent with / in a manner that may not be consistent with} prescribing habits.

Previous **adjunctive therapies** noted in chart review:

- ***

**Falls Risk Assessment**

@LASTFALLSRISKSCREEN@ - provides answers to questionnaire if answered within the past year

@MED@ - current medication list

@AFUTAPPT@ - future appointment(s) scheduled

_______________________________________________

**Cascading Choice Options**

**1: Pharmacist tapering recommendations:** recommend initiating opioid taper, recommend continuing opioid taper, recommend initiating benzodiazepine taper, recommend continuing benzodiazepine taper, and no opioid or BZD taper recommended at this time

**2: Adjunctive therapies:** Acetaminophen, Diclofenac, Duloxetine, Gabapentin, Lidocaine, Pregabalin (Lyrica), OTC counterirritant (Aspercreme, Capzasin), Prednisone, Venlafaxine, Buspirone, Citalopram, Escitalopram, Fluoxetine, Melatonin, Mirtazapine, Sertraline, Trazadone

**3: Pain non-pharm therapies:** rehab/physical therapy, cognitive behavioral therapy, physical exercise, acupuncture, biofeedback & relaxation, massage therapy, stretch therapy, weight loss

**4: Anxiety non-pharm therapies:** mindfulness activities, cognitive behavioral therapy, physical exercise, massage therapy, meditation, stress reduction

**5: Insomnia non-pharm therapies:** sleep hygiene, sleep consolidation, biofeedback & relaxation, cognitive behavioral therapy, meditation, mindfulness activities, stress reduction

**6: Length of therapy:** less than 6 weeks, greater than 6 weeks, *** months, 1 year, greater than 1 year, unknown duration, but at least 6 weeks

**Visual Screenshot Examples of Data Tools Used in Study**

**Figure A:** Dropdown feature allowing common choices of preset phrases


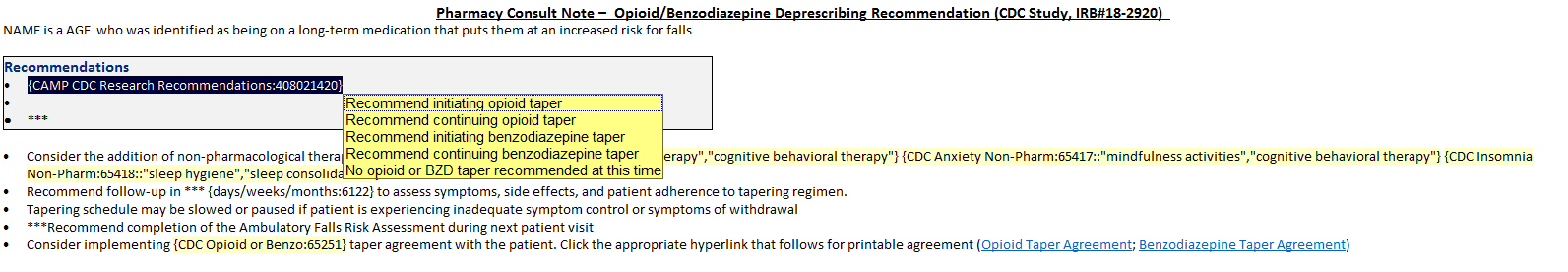


**Figure B:** Template for pharmacy consult note as it appears in EHR


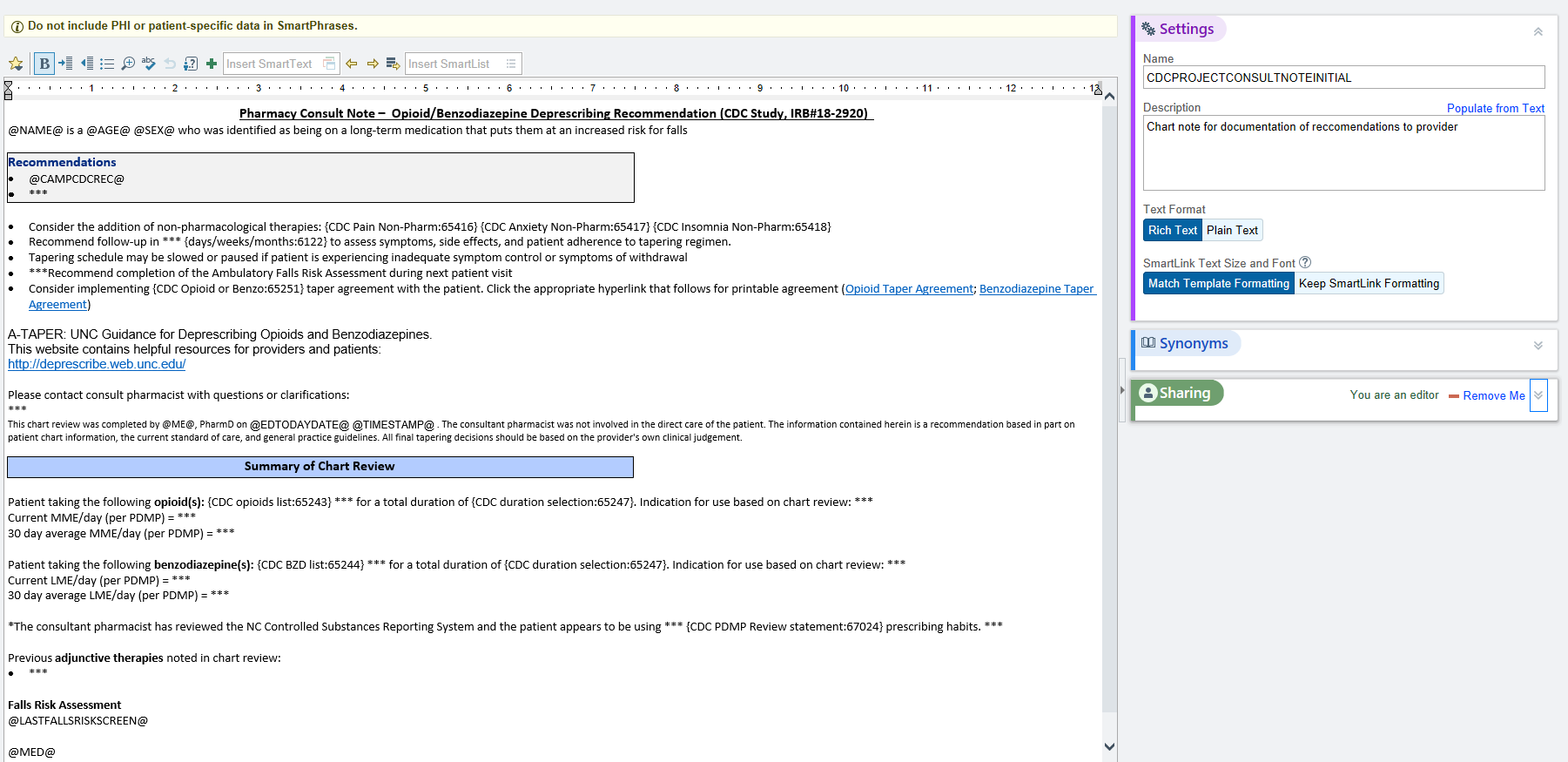

Supplement: ooad034_Supplementary_Data [file ooad034_supplementary_data.docx]
